# ﻿Discovery of a new species of *Synergus* (Hymenoptera, Cynipidae, Synergini) based on morphology and molecular data

**DOI:** 10.3897/zookeys.1193.105756

**Published:** 2024-02-29

**Authors:** Wang Xiudan, Liu Luan, Zeng Yang

**Affiliations:** 1 Laboratory of Insect Behavior and Evolutionary Ecology, College of Life Science and Technology, Central South University of Forestry and Technology, Changsha, Hunan 410004, China Central South University of Forestry and Technology Changsha China

**Keywords:** ASAP, COI, gall wasp, inquilines, integrative taxonomy

## Abstract

A new species of gall inquiline, *Synergusdilatatus***sp. nov.**, is described from Hubei Province, China. Morphological descriptions, photographs and biological information are provided. Mitochondrial cytochrome oxidase (COI) sequences of the new species were newly obtained and a molecular species delimitation analysis of 12 species of *Synergus* performed using the ASAP method recovered 16 molecular operational taxonomic units, providing support for recognition of the new species. The results also highlight a few conflicts between morphological and molecular species delimitations in *Synergus*.

## ﻿Introduction

Cynipids (Hymenoptera: Cynipidae) are mainly known as gall inducers, making various kinds of galls from normal plant tissues that provide food and protection for the growing larvae. However, a small subset of cynipids, termed inquilines, which do not induce galls, live inside galls induced by other species, primarily cynipids, but also gall midges and cecidosid moths (Ronquist 1994; Csóka et al. 2005; Schwéger et al. 2015). Although, as an exception, *Synergusitoensis* Abe, Ide & Wachi, 2011, may induce its own gall in the seed coat of the acorn of Quercus (subgen. Cyclobalanopsis) glauca Thunb. (Abe et al. 2011). All inquiline cynipid wasps from the Holarctic were initially assigned to the tribe Synergini s. l., which was recently subdivided into three tribes (Synergini s. s., Ceroptresini and Diastrophini) by Ronquist et al. (2015) on the basis of molecular and morphological evidence. Synergini s. s. include only the inquiline species from cynipid galls of oaks and *Rhus*. To date, Synergini s. s. consists of six genera: *Agastoroxenia* Nieves-Aldrey & Medianero, *Lithosaphonecrus* Tang, Melika & Bozsó, *Saphonecrus* Dalla-Torre & Kieffer, *Synophrus* Hartig, *Synergus* Hartig and *Ufo* Melika & Pujade-Villar, with *Rhoophilus* Mayr, a genus endemic to South Africa, recently moved to a new tribe according to a recent comprehensive phylogenetic work on global Synergini (Schwéger et al. 2015; Lobato-Vila et al. 2022a). The Chinese Synergini were recently reviewed by Lobato-Vila et al. (2022a). Only four genera were found in mainland China: *Lithosaphonecrus*, *Saphonecrus*, *Synergus* and *Ufo*.

*Synergus* Hartig is presently the most species-rich genus of inquilines with 127 species known worldwide, including 76 species found from the Nearctic and Neotropical regions and 51 from the Palaearctic region. The monophyly of *Synergus* was considered dubious by previous authors, with New World groups considered to be separate genera (Schwéger et al. 2015; Lobato-Vila et al. 2022a). However, the Palaearctic group was considered to be a natural group, so-called “true” *Synergus*, which is distinct from the New World groups based on both morphology and molecular analysis (Pénzes et al. 2012; Lobato-Vila et al. 2022a).

The Eastern Palaearctic (EP) fauna of *Synergus* has been understudied with Pujade-Villar et al. (2014) providing the first revision of 10 species from this region. More recently, Pujade-Villar et al. (2017) described one new species from China. Eight new species and one new synonym were proposed by Schwéger et al. (2015). Lobato-Vila et al. (2020) found one new species from South Korea. Three species from China were described by Lobato-Vila et al. (2021), who also synonymized the recently described species *Synerguschangtitangi* Melika & Schwéger, 2015 with *Synergusdeqingensis* Pujade-Villar, Wang & Chen, 2014. Currently, 23 species of *Synergus* are recorded from the Eastern Palaearctic and mainland China (Lobato-Vila et al. 2022b), which was suspected to harbor an abundant gall wasp fauna due to the diversity of Fagaceae plants in this area.

This article describes a new species of *Synergus* collected from *Quercusvariabilis* in Hubei Province. We use both morphological characters and DNA sequences to support the establishment of a new species and provide morphological descriptions, diagnostic remarks, localities, gall morphology and photographs.

## ﻿Materials and methods

### ﻿Material

Galls collected from late July 2021, in Hubei Province were kept and delivered to the laboratory for artificial breeding in sealed plastic jars with moistened cotton. The adult insects emerged from fine-meshed rearing cages under ambient temperature. All rearing cages were placed in the lab and checked daily for emergence. Emerged adult wasps were captured and preserved in labeled vials with 99% ethanol, stored in a freezer at -80 °C.

### ﻿Morphology

The terminology follows Liljeblad and Ronquist (1998) and Melika (2006) for morphological structures, Ronquist and Nordlander (1989) for forewing venation and Harris (1979) for patterns of cuticular sculpture. Measurements and abbreviations used herein are: F1–F12, first and subsequent flagellomeres; POL (post-ocellar distance), the distance between the inner margins of the posterior ocelli; OOL (ocellar-ocular distance), the distance from the outer edge of a posterior ocellus to the inner margin of the compound eye; and LOL, the distance between posterior and frontal ocelli. The width of the forewing radial cell is measured from the margin of the wing to the Rs vein. Morphological studies were based on the dried and point-mounted specimens. A few specimens were dismembered for detailed photographs of characters and stored in small vials filled with dry cotton. Photographs were taken using a Leica M205C microscope system equipped (Leica Inc., Germany) with a Leica DMC6200 digital camera attached to a computer. Images of the galls were taken with a Nikon D80 ca­mera. All types are deposited in the Insect Collection, Central South University of Forestry and Technology, Changsha, Hunan (**CSUFT**).

### ﻿Molecular data and analyses

Total DNA was extracted from three individuals (destroyed) of the new species which have the same collection data as the types. The insects were washed in sterile water before DNA extraction to avoid cross-contamination. The methods of DNA extraction and PCR cycling conditions were previously described in Zhu et al. (2007). We chose a specific region of the cytochrome oxidase subunit I gene (COI), which was amplified using the primers HCO-2198 and LCO-1490 (Folmer et al. 1994). The barcode region of COI gene showed a clear barcoding gap among species and provided consistency in the phylogenetic topology inferred by mitochondrial and nuclear genes in *Synergus* (Ács et al. 2010).

Forty-five sequences from 12 species representing *Synergus* from the EP as ingroups and three sequences of *Rhoophilusloewi* Mayr (accession numbers: DQ012650, EF486875, EF486876) as outgroups were used for the analyses. The relationship between *Synergus* and remaining genera of Synergini was poorly resolved. Therefore, we chose *Rhoophilus* as an outgroup, which was reconstructed as sister to Synergini (Lobato-Vila et al. 2022a). Three sequences of the new species were newly obtained in this study. The remaining sequences were all downloaded from GenBank, with accession numbers listed in Table 1. Multiple sequence alignment was performed using online MAFFT 7 (https://mafft.cbrc.jp/alignment/server/) (Katoh et al. 2019) under the Q-INS-i algorithm, then adjusted manually after translation into amino acid sequences using MEGA 11 (Kumar et al. 2016). The intraspecific and interspecific genetic divergence values were estimated using a Kimura 2-parameter (Kimura 1980) distance model in MEGA 11. The maximum likelihood tree was constructed in IQ-TREE with 1000 ultrafast bootstrap replicates (Nguyen et al. 2015). The resulting tree file was viewed and adjusted using FigTree v.1.4.3. We also conducted an ASAP (Assemble Species by Automatic Partitioning) analysis to further test the morphological delimitation of the new species (Puillandre et al. 2021). ASAP analysis was conducted using a web interface (https://bioinfo.mnhn.fr/abi/public/asap/asapweb.html) under the Jukes-Cantor (JC69) model. Other parameters were default.

**Table 1.** GenBank accession numbers of haplotype sequences used in species delimitation and their original publications.

Species	GenBank accession numbers	References
*Synergusabei*	KR270552; KR27055; KR270553	Schwéger et al. 2015
*Synergusbelizinellus*	KR270554; KR270555; KR270556; MW274094	Schwéger et al. 2015; Lobato-Vila et al. 2022a
*Synerguscastaneus*	KC533845; KC533846; KC533847; KC533848; KC533849; KC533850	Bernardo et al. 2013
*Synerguschinensis*	EF486890MW274096; MW274097; MW274095	Ács et al. 2010; Lobato-Vila et al. 2022a
*Synergusformosanus*	KR270547; KR270546; KR270545	Schwéger et al. 2015
*Synergusgifuensis*	LC272567	Ide et al. 2018
*Synergusishikarii*	KR270548; KR270550; KR270549; MW274110	Schwéger et al. 2015; Lobato-Vila et al. 2022a
*Synergusitoensis*	LC272566	Ide et al. 2018
*Synergusjaponicus*	KR270560; LC272564; EF486926; EF486927	Ács et al. 2010; Schwéger et al. 2015; Ide et al. 2018
*Synerguskhazani*	KR270557; KR270558	Schwéger et al. 2015
*Synergussymbioticus*	KR270540; KR270544; KR270542; KR270541; KR537438; KR537437; KR537436; KR270543; MW274123	Schwéger et al. 2015; Lobato-Vila et al. 2022a
*Synergusxiaolongmeni*	EF486968	Ács et al. 2010
*Synergusdilatatus* sp. nov.	OQ850003; OQ850004; OQ850005	In this study

## ﻿Results

**Figure 1.**
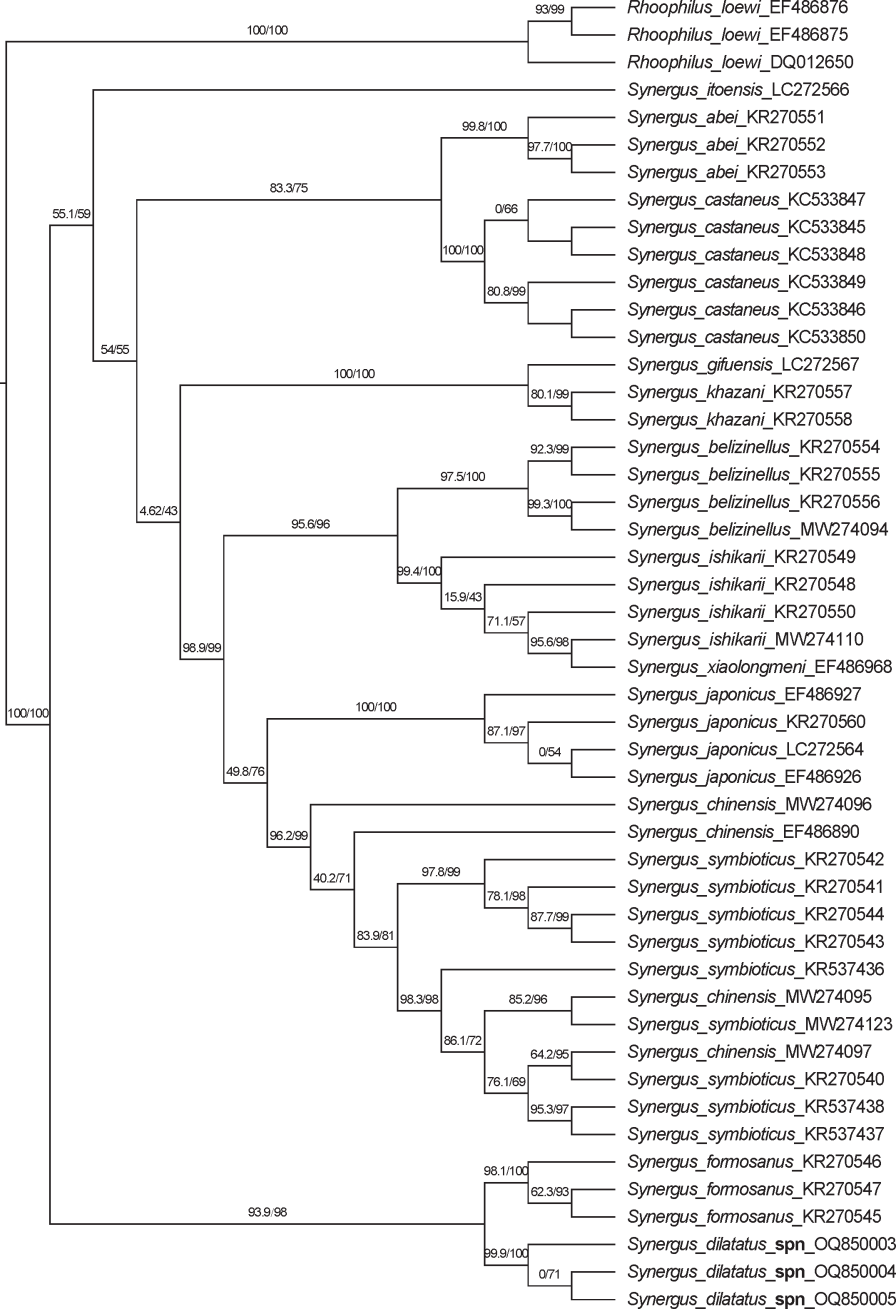
Maximum-likelihood tree derived from COI sequences of 45 species of *Synergus* from the EP, with 1000 bootstrap replicates. Numbers on nodes are SH-aLRT and bootstrap values.

The gall samples were collected in the summer of 2021 in Hubei Province. Seventeen females and six males emerged in succession during August of that year. Three of them were destroyed for DNA extraction, and eight were mounted as type specimens. Reminders were preserved in 99% ethanol for further genomic study. Assessment of morphological characters suggested that the emerging inquiline species belongs to the genus *Synergus*. The target 1062 base pair (bp) fragment of COI was successfully amplified from three individuals drawn from two different galls. The new species is morphologically similar to the known species, *S.formosanus*, discovered from Taiwan; however, the genetic distance of COI between those two species reached 9% (Suppl. material 1: appendix S1). The analysis of twelve morphospecies of *Synergus* from the EP shows that all morphological species are monophyletic, except *S.chinensis*, *S.symbioticus*, *S.ishikarii* and *S.xiaolongmeni* (Fig. 1). For the ASAP analysis, ten best partitions were found, with 10 to 30 molecular operational taxonomic units (MOTUs) estimated for the 45 samples (Suppl. material 2: appendix S2). The number of species predicted based on the partitions ranked first is 16, suggesting that the diversity of *Synergus* from the EP may be underestimated by the current morphology-based species classification. However, all ten partitions strongly supported the validity of the new species.

### ﻿Taxonomy

#### ﻿ Synergus dilatatus sp. nov.

Taxon classificationAnimaliaHymenopteraCynipidae

885392BA-5D96-5F17-8C1D-C603BF5871E4

https://zoobank.org/4F232E05-A89A-4F73-9D90-1B364818CA0C

Figs 2–9 , 10–17 , 18

##### Type materials.

***Holotype*.** China ·♀, Hubei Prov., Suizhou County; 31.69°N, 113.38°E (DD); galls coll. 30 Jul. 2021, ex. Aug. 2021; Yang Zeng leg.; from twigs on *Quercusvariabilis*. ***Paratypes*.** China ·5 ♀ & 2 ♂, same data as the holotype.

##### Diagnosis.

The new species is similar to *S.formosanus* Schwéger & Melika, 2015. *Synergusdilatatus* sp. nov. but differs from the latter by female head dark red to black (orbits, malar space and a band above clypeus chestnut brown in *S.formosanus* female); ventral margin of malar space expanded into a wide and recurved lamina (ventral margin not or slightly expanded in *S.formosanus*); medial mesoscutal line restricted to posterior half of mesoscutum (on posterior two-thirds of mesoscutum in *S.formosanus*); and parapsidal line absent (present in *S.formosanus*). The attacked galls of the new species, whose surface is covered with dense tomentum, is without tubercles or spots (the attacked galls of *S.formosanus* are covered with small raised tubercles and purple spots).

In the key to species of *Synergus* from the EP presented by Schwéger et al. (2015: 458), *S.dilatatus* sp. nov. follows item 15 and the key should be adapted as described below:

**Figures 2–9.**
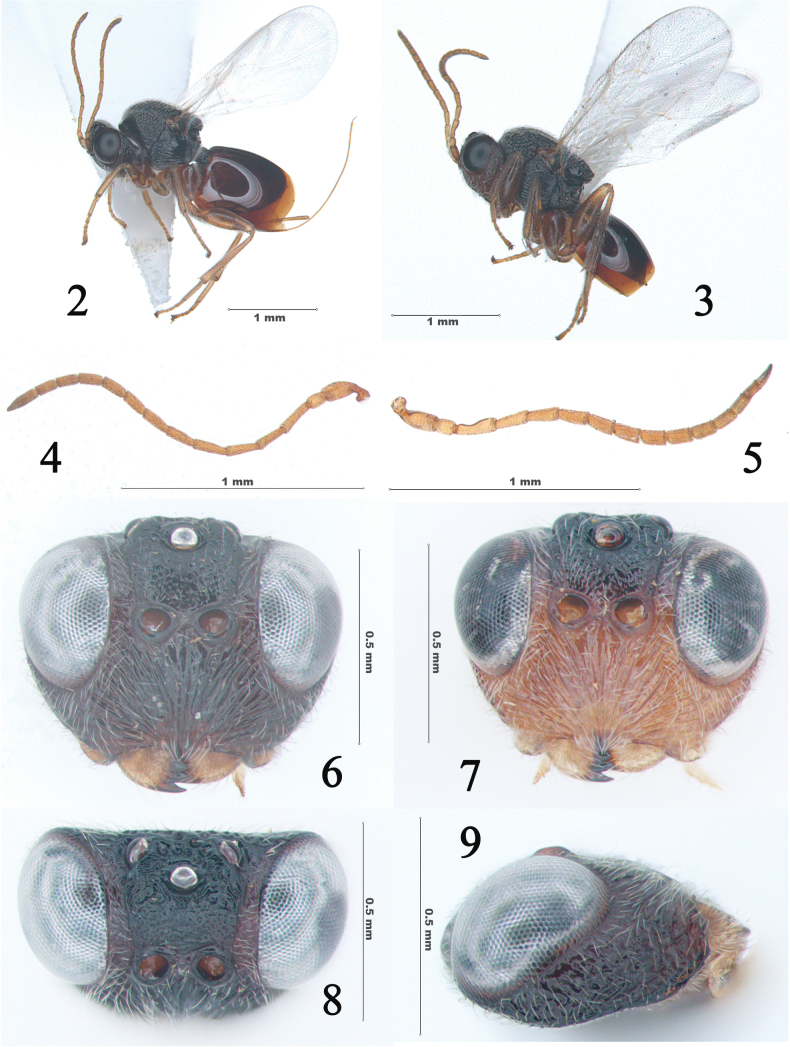
*Synergusdilatatus* sp. nov. **2** general habitus (♀) **3** general habitus (♂) **4** antenna (♀) **5** antenna (♂) **6** head in anterior view (♀) **7** head in anterior view (♂) **8** head in dorsal view (♀) **9** head in lateral view (♀).

**Table:** 

15	Postgena with dense white setae on lower half; median mesoscutal line strongly impressed, complete, reaches anterior margin of mesoscutum	***S.symbioticus* Schwéger & Melika, 2015**
–	Postgena mostly glabrous on lower half; median mesoscutal line strongly impressed only in posterior half, extending to 2/3 of mesoscutum length, never complete	**16**
16	Ventral margin of malar space expanded into a wide and recurved lamina; medial mesoscutal line restricted to posterior half of mesoscutum; parapsidal line absent	***S.dilatatus* sp. nov.**
–	Ventral margin not or slightly expanded; medial mesoscutal line on the posterior two-thirds of mesoscutum; parapsidal line present	***S.formosanus* Schwéger & Melika, 2015**

##### Description.

**Female.** Body length 2.4–2.8 mm (*N* = 14).

***Color***: Head reddish dark brown to black, except mouthparts yellowish brown; antenna yellowish to light brown. Mesosoma black. Legs yellowish or light brown. Wings hyaline with slightly darker coxae, veins pale yellowish. Metasoma with 1^st^ tergite dark reddish brown and rest of metasoma reddish brown, ventral spine of hypopygium light brown.

***Head*** (Figs 6, 8, 9): Nearly trapezoid, 1.2 times as wide as high in front view, distinctly broader than mesosoma, 2.6 times as broad as long in dorsal view. The central area of frons elevated and delimited by two dull lateral frontal carinae, the surface finely coriaceous, puncticulate, and sparsely setose; eyes 1.6 times as high as wide; height of eye 2.0 times as high as length of malar space (Fig. 6). Lower face, clypeus and malar space with dense, long, white setae, except area under antennal sockets sparsely setigerous. Lower face with distinct striae radiating from clypeus and reaching basal margin of compound eye and antennal toruli. Ventrolateral corner of malar space expanded as a wide and recurved lamina. Clypeus barely impressed, covered with long appressed setae; anterior tentorial pit small, distinct; clypeo-pleurostomal line and epistomal sulcus absent. Transfacial distance about the same as the height of the compound eye; distance between torulus and compound eye subequal to diameter of toruli and 1.67 times as great as the distance between toruli (Fig. 6). Vertex rugulose around the anterior ocellus, and from interocellar area to occiput sparsely foveolate; POL: OOL: LOL= 7: 3: 3; OOL and LOL as long as diameter of frontal ocellus (Fig. 8). Postgena glabrous, gular sulcus and posterior tentorial pits distinct, with slightly white setae close to posterior tentorial pits. Gena not broadened posterior to eye, and areolate-rugose (Fig. 9). Antenna filiform with 12 flagellomeres, slightly thickened toward apex; pedicel 1.3 times as long as broad, not broadened apically, with dense and short pubescence; placoid sensillae slightly visible on flagellar segments F3-F12. F1 about 1.2 times the length of F2 and as long as F3. Last flagellar segment 3.75 times as long as wide. Relative length from pedicel to F12: 14:12:14:14:13:13:11:12:10:9:9:15 (Fig. 4).

**Figures 10–17.**
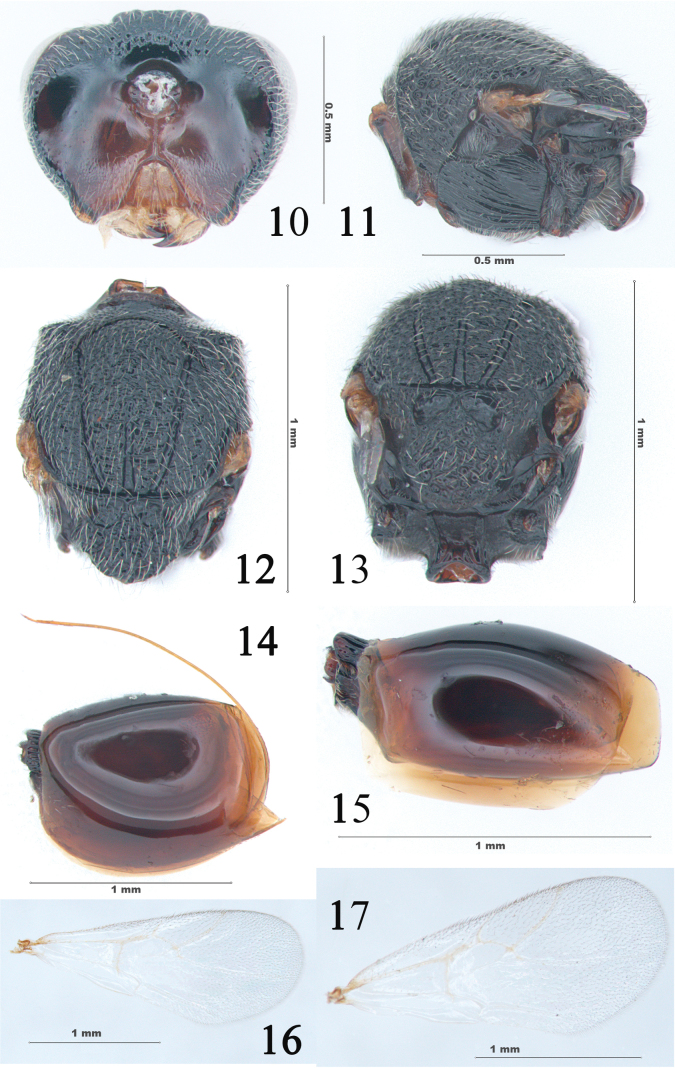
*Synergusdilatatus* sp. nov. **10** head in posterior view (♀) **11** mesosoma in lateral view (♀) **12** mesosoma in dorsal view (♀) **13** propodeum in dorsal view (♀) **14** metasoma in lateral view (♀) **15** metasoma in lateral view (♂) **16** fore wing (♀) **17** fore wing (♂).

***Mesosoma***: slightly longer than high and 1.3 times as long as wide (Fig. 11). Pronotum slightly narrowed medially, median length 1/3 length of outer lateral margin; sides of pronotum angled in dorsal view (Fig. 12). Pronotum puncticulate medially and laterally with deep punctures. Propleuron alutaceous, with parallel longitudinal striae; pronotal lateral carina present. Mesoscutum slightly broader than long measured at anterior tip of tegulae; surface areolate-rugose, covering with dense white setae. Notaulus complete, with smooth bottom. Anterior parallel line invisible; distinct parascutal carina present; parapsidal lines hardly traceable; median mesoscutal line broadened posteriorly and never extending to anterior half of mesoscutum (Fig. 12). Dorsoaxillar area coriaceous, with numerous long hairs; lateroaxillar area joined by dorsoaxillar area at an acute angle. A median carina separates mesoscutellar foveae into two parts which are as long as wide, bottom hairless and coriaceous, and distinctly delimited posteriorly. Mesopleuron hairless laterally except the lower edge densely setae, fully covered with parallel longitudinal striae. Metapleural sulcus reaching posterior margin of mesopleuron in upper 1/3 of its height (Fig. 11). Mesopleural triangle densely pubescent. Propodeum uniformly coriaceous, pubescent; lateral propodeal carinae distinct, anteriorly flat and posteriorly thin, slightly curved; median propodeal area delicately coriaceous without setae, with few rugae posteriorly. Metanotal trough smooth, pubescent; propodeal spiracle transversely ovate. Nucha very small, with distinct longitudinal parallel rugae (Fig. 13).

***Forewing*** (Fig. 16): Hyaline, margin with cilia; radial cell partially closed, 2.4 times as long as broad; R1 nearly straight and reaching wing margin, Rs curved distally, distinctly reaching wing margin; areolet small but distinct; Rs + M inconspicuous, not reaching basal vein.

***Metasoma***: About as long as head and mesosoma combined and 1.4 times as long as high; petiole sulcate; syntergite almost completely covering remaining tergites, surface smooth and mainly glabrous, with very few white setae anterolaterally, posterodorsal area with lateral patch of sparse setae and indistinct micropunctures, not extending onto lateral sides. Subsequent tergites and hypopygium micropunctate; prominent part of the ventral spine of hypopygium small, smooth with few short setae distally (Fig. 14).

**Male** (Figs 3, 5, 7, 15, 17). Body length: 1.5–2.3 mm (*N* = 4). Lower face and mouthparts yellowish brown; frons, vertex and occiput reddish brown to black (Fig. 7). Antenna with 13 flagellomeres, scape length 3 times as long as wide; pedicel 1.6 times as long as broad, F1 strongly curved medially and broadened apically, 1.6 times as long as pedicel and 1.2 times as long as F2; relative length of F1-13: 13:11:11:10:9:9:8:8:8:8:7:7:8; placoid sensilla slightly visible on F3–F13, increasing in number per segment distally (Fig. 5). Metasoma distinctly shorter than head and mesosoma combined (Fig. 3) and slender than female in lateral view (Fig. 15). Otherwise, as described for female.

##### Biology and galls.

**Figures 18.**
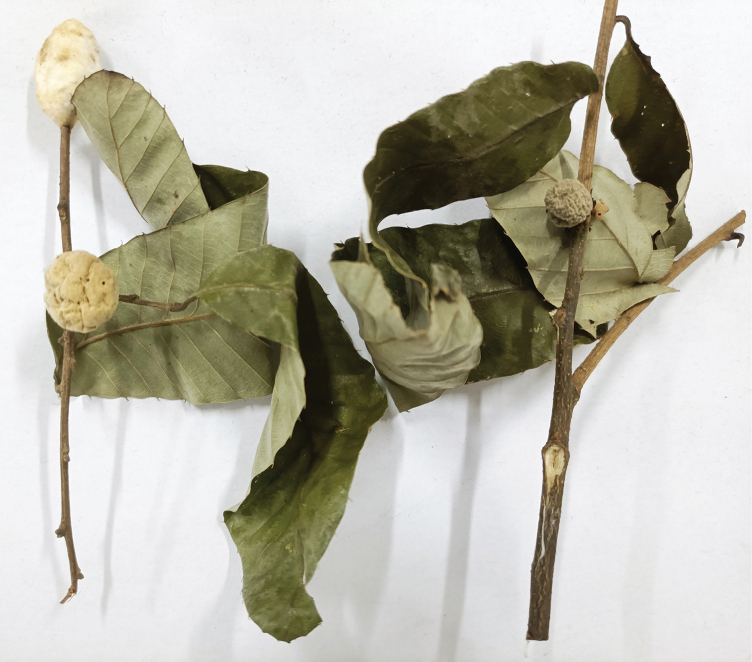
General appearance of *Synergusdilatatus* sp. nov. galls on twigs of *Quercusvariabilis*.

All specimens were reared from two juicy, green, young galls resembling fruits, collected from shoots of *Quercusvariabilis* on July 30, 2021. Galls are ball-shaped, with diameter 14–22 mm; surface smooth, covered with dense tomentum, without tubercles or spots. The galls became wrinkled and shriveled after inquilines emerged in late August (Fig. 18). Unfortunately, no gall makers emerged, so the species that formed the galls remains unknown.

##### Distribution.

Known only from Hubei Province (China).

##### Etymology.

The species is derived from Latin word “*dilatatus*” meaning dilated, extended, referring to the dilated gena of the new species.

## ﻿Discussion

Species delimitation is difficult for inquilines based on morphology. For example, *Synergusjaponicus* and *S.gifuensis* Ashmead, 1904 were treated as two different forms of *Synergusjaponicus* based on similarity in morphology, but are distinct in life cycles and impact on the gall structures (Masuda 1959; Abe 1990; Abe 1992; Pujade-Villar et al. 2002). DNA barcoding as a complementary taxonomic approach has considerable utility for inquilines and the status of some Western Palaearctic *Synergus* species has been questioned based on disagreement between morphospecies and MOTUs (Ács et al. 2010). In this study, the ASAP method was used in support of the new species, *Synergusdilatatus* sp. nov., with an integrative approach combining morphological and molecular data, and the results were consistent.

However, among the 12 morphospecies analysed here, 16 MOTUs were chosen by the best partition. The main differences are: (1) *S.belizinellus* is divided into two MOTUs; (2) *S.xiaolongmeni* and *S.ishikarii* are merged into one MOTU; and (3) *S.chinensis* and *S.symbioticus* are divided into four MOTUs, which is consistent with the results of Lobato-Vila et al. (2022b). Those mismatches between morphological and MOTU-based identification are unsurprising given the similar situation in the WP groups (Ács et al. 2010). Considering the phenotypic similarity shared by those problematic species (*S.xiaolongmeni* and *S.ishikarii*; *S.chinensis* and *S.symbioticus*) (Schwéger et al. 2015), the current morphological characters used to distinguish species should be reviewed by integrating molecular data.

## Supplementary Material

XML Treatment for Synergus dilatatus

## ﻿Additional information

### Author ORCIDs

Wang Xiudan https://orcid.org/0000-0002-6444-9830

Liu Luan https://orcid.org/0009-0002-1585-7587

Zeng Yang https://orcid.org/0000-0003-4892-2007

### Data availability

All of the data that support the findings of this study are available in the main text or Supplementary Information.

## Supplementary materials

Supplementary material 1 appendix S1 Data type xls Explanation note Genetic distance data of 12 species of EP*Synergus*. https://binary.pensoft.net/file/994954This dataset is made available under the Open Database License (http://opendatacommons.org/licenses/odbl/1.0/). The Open Database License (ODbL) is a license agreement intended to allow users to freely share, modify, and use this Dataset while maintaining this same freedom for others, provided that the original source and author(s) are credited.Wang Xiudan, Liu Luan, Zeng Yang

Supplementary material 2 appendix S2 Data type jpg Explanation note Boxed subsets resluting from ASAP analyses. https://binary.pensoft.net/file/994955This dataset is made available under the Open Database License (http://opendatacommons.org/licenses/odbl/1.0/). The Open Database License (ODbL) is a license agreement intended to allow users to freely share, modify, and use this Dataset while maintaining this same freedom for others, provided that the original source and author(s) are credited.Wang Xiudan, Liu Luan, Zeng Yang
