# Supplementary figures and images for: ﻿Discovery of a new species of Synergus (Hymenoptera, Cynipidae, Synergini) based on morphology and molecular data
Source: Zookeys. 2024 Feb 29;1193:81–94. doi: 10.3897/zookeys.1193.105756 (PMC10921061; doi:10.3897/zookeys.1193.105756)

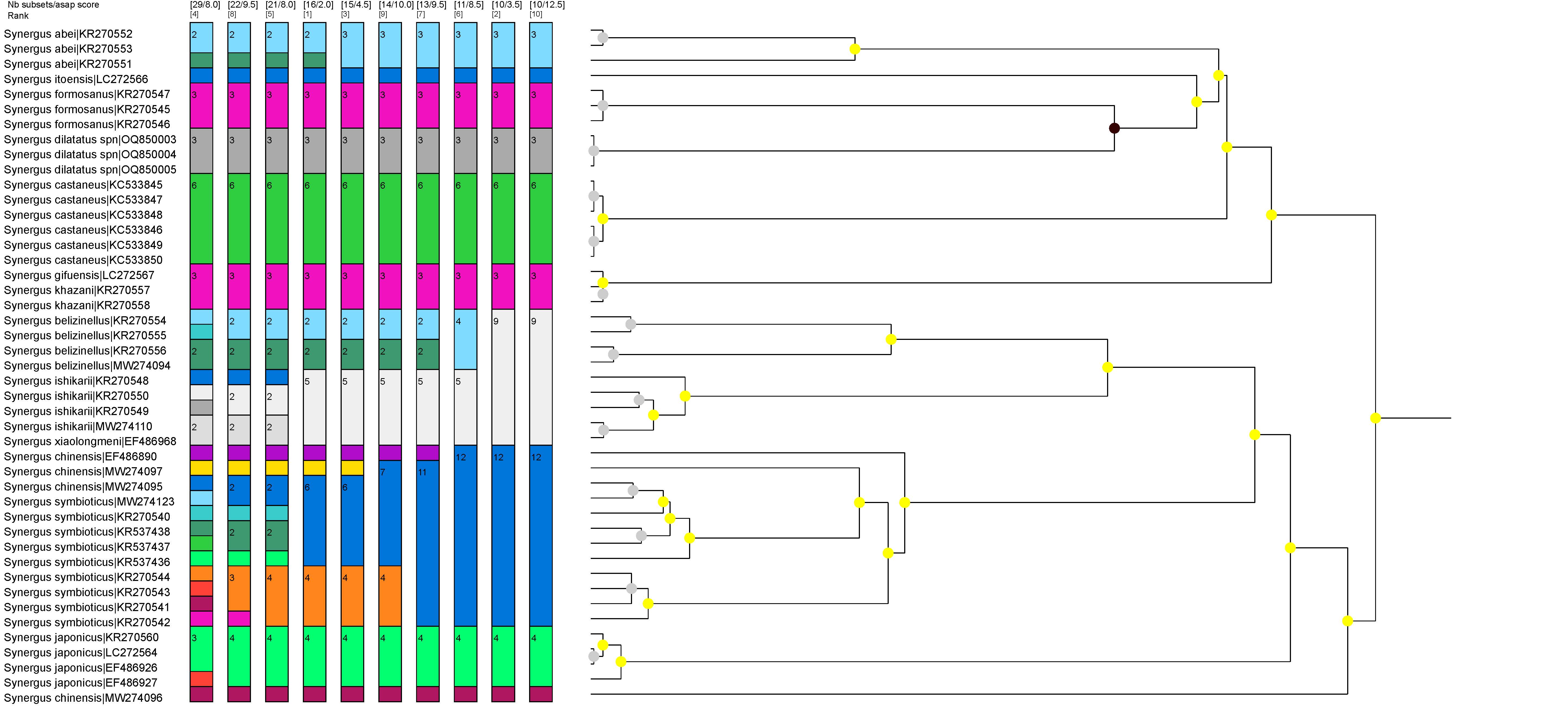

Supplement: Supplementary material 2 — appendix S2 [file zookeys-1193-081_article-105756__-s002.jpg]
